# Four years of the primary care-internal medicine continuity of care unit in the health region of Guadalajara/Cuatro años de la Unidad de Continuidad Asistencial Primaria-Interna (UCAPI) en el área sanitaria de Guadalajara

**Published:** 2012-05-29

**Authors:** J.M. Machín Lázaro, A. Pereira Juliá, M. Rodriguez Zapata, A. Bárcena Marugán, E. Martín Echevarría, M.L. Díez de Andrés

**Affiliations:** Department of Internal Medicine—PCIM CCU, University Hospital of Guadalajara, Spain; Department of Internal Medicine—PCIM CCU, University Hospital of Guadalajara, Spain; Department of Internal Medicine—PCIM CCU, University Hospital of Guadalajara, Spain; Department of Medicine, University of Alcalá, Spain; Primary Care Administration for Guadalajara, Spain; Department of Internal Medicine—PCIM CCU, University Hospital of Guadalajara, Spain; Cervantes Health Centre, Guadalajara, Spain

**Keywords:** comorbidity, chronic disease, continuity of patient care, comorbilidad, enfermedad crónica, continuidad de cuidados

## Introduction

The Primary Care Internal Medicine Continuity of Care Unit (PCIM CCU) was established to address gaps identified in the care provided for complex chronic patients.

## Description of the project

The PCIM CCU was set up in June 2006 from the Department of Internal Medicine at the University Hospital of Guadalajara, reaching out to primary care (PC), with the aim of achieving collaboration and coordination between the two levels of care and avoiding the sense of imposition that can be felt in primary care with regards all that comes from the hospital. To this end, several meetings were held between the medical management for primary care, the coordinators of the various primary care teams and the internists responsible for the PCIM CCU prior to the launch of this pilot project.

**Figure 1.**
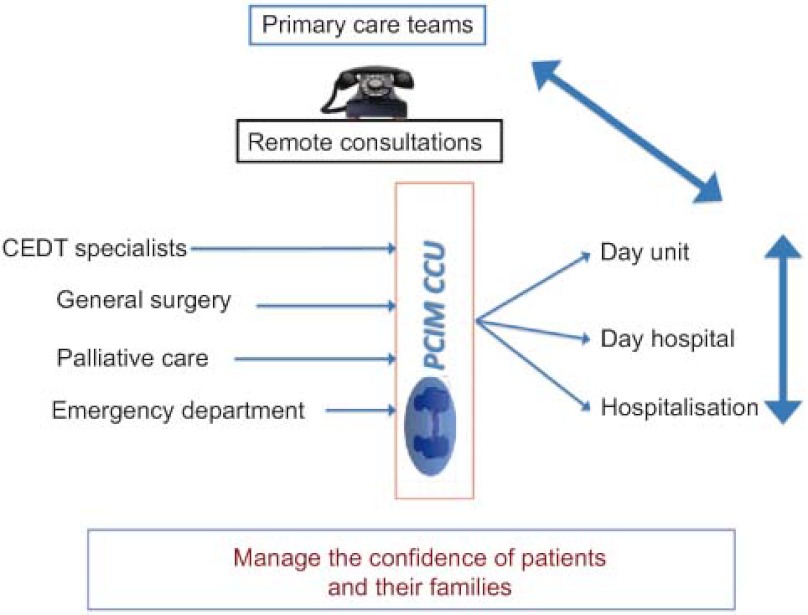
Flow chart of the care pathway in the PCIM CCU. Note: CEDT: Spanish acronym for Centre for Specialities, Diagnosis and Treatment. It is a building in which both primary care physicians and specialists work.

The objective of the project was to facilitate continuity of care for patients with complex chronic conditions and those with multiple diseases as well as cases in which diagnosis must not be delayed. The methodology for the project (see Figure 1) was similar to that described in the guidelines “*Unidades de Pacientes Pluripatológicos: Estándares y recomendaciones*” [1] published by the Spanish Ministry of Health and Social Policy: namely, a consultant internist is assigned to consistently work with three to four health centres in the Guadalajara Health Region, and he/she is available on a mobile phone (provided by the health organisation) from 9 am to 7 pm, Monday to Friday, or by email. Additionally, these internists hold sessions in the health centres once a month. The objectives of these consultations are: to solve queries arising when a patient is seen in primary care, to request an appointment (within a maximum of 72 hours) with the internist, to discuss diagnostic test results (ECG, radiographies, etc.) and to request patient reports, thereby avoiding unnecessary check-ups and appointments.

Medical staff involved: one internist until February 2010, and subsequently three.

Infrastructure: the intention was to use already existing personnel (nursing and administrative staff) and facilities; for this, a hospital ward was restructured, being used jointly with the medical and surgical short stay unit (SSU), with the following facilities: a consultation room, day hospital area and conventional inpatient beds.

The initial catchment population of the PCIM CCU, when there was only one internist, was 58,479 people under the care of three health centres (two urban centres, within 10 minutes on foot from the hospital and a rural one 85 km from the hospital). In February 2010, the project was extended to seven health centres, increasing the catchment population to 134,613 and involving three internists.

## Results

The data on the activity of the PCIM CCU in its five-years of operation are reported below in relation to the various areas of the unit’s work.

### Remote consultations by mobile/e-mail

A total of 1892 telephone calls were made to the internists, of which only 762 resulted in new patient referrals to the PCIM CCU; the remaining calls were queries that were resolved at the time.

### Consultations

A total of 1989 patients were seen. The ratio of follow-up to first appointments ranged from 0.81 to 1.95; that is, there were fewer than two check-ups for every new patient seen.

The characteristics of patients seen in consultations were as follows [2]: patients with multiple diseases (33%), symptomatic chronic patients (24%), cases in which diagnosis must not be delayed (32%) and non-specific conditions (11%). Of the 762 new patients, we managed to avoid admission in 16% of cases (124 patients) and in 29.66% (228) we avoided patients going to the hospital emergency department, thanks to the support of the Day Hospital and self-management of consultations and beds by the internists leading the PCIM CCU.

### Day Hospital

It has four arm chairs for diagnostic and therapeutic procedures. During the study period a total of 676 procedures were carried out, of which the most common were: blood sample collection and parenteral treatments, in particular iron infusion. Other procedures included: paracentesis, thoracocentesis, and lumbar punctures.

### Hospitalisation

The ward being shared with the SSU, eight beds were assigned to the PCIM CCU. Patients could be admitted either directly from specialist appointments or the Day Hospital, or from the hospital emergency department, provided that they were from the catchment area of the health centres participating in the PCIM CCU programme.

The care indices in 2010 were better that those generated by conventional hospitalisation under the care of the Department of Internal Medicine. In particular, the risk-adjusted average length of stay index was 0.93, that is, lower than the overall value for the Health Service of 1 and that of the Department of Internal Medicine itself, which was 1.01. Further, the impact of the care provided to patients managed by the PCIM CCU is -171, that is, we have saved 171 hospital bed stays, while traditional healthcare under the Department of Internal Medicine used, for the same year, 156 more hospital bed days (impact of +156). Put another way, if we estimate the impact as a function of the standard average length of stay by diagnostic related group (DRG) in the Spanish National Health System, in 2010, the PCIM CCU managed to reduce hospitalisation, saving 171 hospital bed days, while the Department of Internal Medicine oversaw the use of 156 bed days that could have been avoided.

## Conclusions

The good outcomes reported are the result of a relatively new approach to care that requires changes in the way of working in both the hospital and primary care. This study demonstrates that the optimisation of hospital resources is feasible through real, effective and personalized coordination between primary care and internal medicine specialists. Despite the fact that healthcare indices generated already indicate benefits of this way of working, further research should be conducted to assess whether it has an impact on the mortality or the readmission rate of patients with multiple chronic diseases.

## Conference abstract Spanish

## Introducción

La Unidad de Continuidad Asistencial Primaria-Interna (UCAPI) se crea por los vacíos existentes en el proceso asistencial a pacientes crónicos complejos.

## Descripción de la experiencia

La UCAPI nace en junio de 2006 desde el Servicio de Medicina Interna del Hospital Universitario de Guadalajara, extendiéndose hacia Atención Primaria (AP) siempre con la filosofía de colaboración y coordinación entre ambos niveles asistenciales, evitando la sensación de imposición que puede generar en Atención Primaria todo aquello que venga del hospital; para ello, se mantuvieron varias sesiones conjuntas con la Dirección Médica de Primaria, los coordinadores de los distintos Equipos de Atención Primaria y los internistas responsables de la UCAPI antes de la puesta en marcha de este proyecto. Su objetivo es facilitar la continuidad asistencial de los pacientes crónicos complejos, de pacientes con pluripatología y de pacientes en fase diagnóstica no demorable.

La metodología de trabajo (ver figura 1) es similar a la que se recoge en el libro “Unidades de Pacientes Pluripatológicos: Estándares y recomendaciones” [1] editado por el Ministerio de Sanidad y Política Social: a cada 3–4 Centros de Salud del Área Sanitaria de Guadalajara se les asigna un internista consultor, que siempre será el mismo, al que se puede acceder mediante móvil corporativo en horario de 9 a 19 horas de lunes a viernes o mediante e-mail, además de las sesiones presenciales que los internistas tendrán en los Centros de Salud una vez al mes. La consultoría tiene estos fines: dudas que surgen en AP mientras se atiende a un paciente, solicitar una cita con el internista que tendrá una demora acordada máxima de 72 horas, comentar pruebas diagnósticas (ECG, radiografías) o solicitar informes sobre pacientes, evitando así revisiones y citaciones burocráticas.

Personal facultativo: un internista hasta febrero de 2010, cuando ya se amplía con tres.

Infraestructura: era deseable emplear personal (de enfermería y administrativo) y material ya existentes; para ello, se reconvierte una planta hospitalaria que se comparte con la Unidad de Corta Estancia (UCE) médica y quirúrgica, donde se ubicará: consulta, hospital de día y hospitalización convencional.

El área que se cubre con la UCAPI constaba inicialmente, cuando estaba sólo un internista, de 3 Centros de Salud que atienden a 58.479 tarjetas sanitarias (dos Centros urbanos, a diez minutos a pie del hospital, y otro, rural a 85 km del hospital). En febrero de 2010 se amplía a 7 Centros, lo que hace un total de 134.613 tarjetas y 3 internistas.

## Resultados

Los datos de la actividad realizada en este tiempo, 5 años, se divide entre los distintos dispositivos con los que cuenta la UCAPI, a saber:

### Consultoría-móvil-mail

se reciben 1892 llamadas a los móviles de los internistas, de las cuales únicamente 762 son nuevas derivaciones de pacientes para ser atendidos en la UCAPI; el resto son dudas que se resuelven sobre la marcha.

### Consulta

se han atendido un total de 1989 pacientes. El índice de consultas sucesivas /primeras es de 0,81 a 1,95; es decir, apenas se generan 2 revisiones por cada paciente nuevo que se atiende.

Descripción de los pacientes atendidos en la consulta [2]: criterios de pluripatología (33%), crónicos sintomáticos (24%), diagnóstico no demorable (32%) y síndromes abiertos (11%). De los 762 pacientes nuevos, en el 16% (124) se ha podido evitar el ingreso y en un 29,66% (228) se ha logrado evitar que acudan a la urgencia hospitalaria, gracias al apoyo del Hospital de Día y la autogestión de la consulta y de las camas por parte de los internistas responsables de la UCAPI.

### Hospital de Día

consta de 4 sillones para procedimientos diagnóstico-terapéuticos. Se realizan en este tiempo 676 procedimientos; los más numerosos: extracciones de analíticas y tratamientos parenterales, concretamente el hierro. Otros procesos realizados son: paracentesis, toracocentesis y punciones lumbares.

### Hospitalización

al ser una planta compartida con la UCE, se han asignado 8 camas. El origen de los pacientes que ingresan puede ser bien directamente de la consulta u Hospital de Día, bien de la Urgencia hospitalaria siempre que los pacientes pertenezcan a los Centros de Salud adscritos al programa de la UCAPI. Los índices asistenciales generados en el año 2010 resultaron más favorables que los generados por la hospitalización convencional en Medicina Interna con un índice de Estancia Media Ajustado en 2010 de 0,93, es decir, inferior a la norma del Servicio de Salud, que es 1, y del propio Servicio de Medicina Interna, que es de 1,01. Además, el impacto que tiene la asistencia a los pacientes ingresados en la UCAPI es de -171, es decir, se han ahorrado 171 estancias hospitalarias, mientras que la asistencia tradicional de Medicina Interna empleó, para el mismo año, 156 estancias hospitalarias más (Impacto de + 156). Esto es, si se calcula el impacto en función del estándar de estancia media por GRD (grupo relacionado de diagnósticos) del Sistema Nacional de Salud, la UCAPI logra ahorrar en su asistencia 171 estancias hospitalarias, mientras que Medicina Interna podría haber evitado 156 estancias en el año 2010.

## Conclusiones

Los datos asistenciales pertenecen a una forma asistencial reciente que obliga a cambiar mentalidades de trabajo en hospital y en Atención Primaria. Este trabajo demuestra la posible optimización de recursos hospitalarios a través de una coordinación real, efectiva y personalizada entre Atención Primaria y Medicina Interna. A pesar de que los índices asistenciales generados son favorables a esta nueva forma de trabajar, todavía queda por conocer si esto incidirá en la tasa de mortalidad o de reingresos de los pacientes con patologías múltiples y crónicas.
